# How the kidney regulates magnesium: a modelling study

**DOI:** 10.1098/rsos.231484

**Published:** 2024-03-20

**Authors:** Pritha Dutta, Shervin Hakimi, Anita T. Layton

**Affiliations:** ^1^ Department of Applied Mathematics, University of Waterloo, Waterloo, Ontario N2L 3G1, Canada; ^2^ Department of Biology, University of Waterloo, Waterloo, Ontario N2L 3G1, Canada; ^3^ Cheriton School of Computer Science, University of Waterloo, Waterloo, Ontario N2L 3G1, Canada; ^4^ School of Pharmacology, University of Waterloo, Waterloo, Ontario N2L 3G1, Canada

**Keywords:** magnesium homeostasis, electrolyte transport, renal transport, sex differences

## Abstract

The kidneys are crucial for maintaining Mg^2+^ homeostasis. Along the proximal tubule and thick ascending limb, Mg^2+^ is reabsorbed paracellularly, while along the distal convoluted tubule (DCT), Mg^2+^ is reabsorbed transcellularly via transient receptor potential melastatin 6 (TRPM6). TRPM6 and other renal transporter expressions are regulated by sex hormones. To investigate renal Mg^2^ handling, we have developed sex-specific computational models of electrolyte transport along rat superficial nephron. Model simulations indicated that along the proximal tubule and thick ascending limb, Mg^2+^ and Na^+^ transport occur parallelly, but they are dissociated along the DCT. In addition, our models predicted higher paracellular Mg^2+^ permeability in females to attain similar cortical thick ascending limb fractional Mg^2+^ reabsorption in both sexes. Furthermore, DCT fractional Mg^2+^ reabsorption is higher in females than in males, allowing females to better fine-tune Mg^2+^ excretion. We validated our models by simulating the administration of three classes of diuretics. The model predicted significantly increased, marginally increased and significantly decreased Mg^2+^ excretions for loop, thiazide and K-sparing diuretics, respectively, aligning with experimental findings. The models can be used to conduct *in silico* studies on kidney adaptations to Mg^2+^ homeostasis alterations during conditions such as pregnancy, diabetes and chronic kidney disease.

## 1. Introduction

Magnesium, the second most abundant intracellular cation, is an important cofactor for numerous biological processes, including protein synthesis, nucleic acid stability and neuromuscular excitability. However, the clinical significance of Mg^2+^ has only been properly acknowledged in recent years. As such, Mg^2+^ was once referred to as ‘the forgotten electrolyte’ [1]. Extracellular magnesium is tightly regulated, with plasma (Mg^2+^) maintained relatively constant between 1.6 and 2.3 mg/dl [2] under normal physiological conditions in humans. Almost all of the body’s Mg^2+^ (approximately 99%) is stored either in bone or within cells [2]. The normal daily Mg^2+^ intake of humans averages around 300 mg, about half of which is absorbed by the intestine [3].

Together with the intestine, the kidneys have an important role in maintaining Mg^2+^ homeostasis. About 70% of the circulating Mg^2+^ is non-protein bound and is thus filtered by the glomerulus, accounting for 2,400 mg in humans. Much of our understanding of Mg^2+^ handling in the various nephron segments has been derived from micropuncture and microperfusion studies in rodent nephrons [4,5]. More recently, genetic studies have expanded our knowledge about the protein mediators of Mg^2+^ transport [6]. The kidney reabsorbs 95–98% of the filtered Mg^2+^ primarily along three nephron segments: the proximal tubule (15–25%), cortical thick ascending limb (cTAL) (60–70%) and distal convoluted tubule (DCT) (up to ~23%) [7]. In the rat kidney, along both the proximal tubule and cTAL, Mg^2+^ is reabsorbed via the paracellular pathway, driven by a favourable electrochemical gradient that arises primarily from the active transport of Na^+^. Along the DCT, Mg^2+^ is reabsorbed transcellularly, mediated by the transient receptor potential melastatin 6 (TRPM6) that is expressed along the apical membrane, and extruded from the cell by the Na^+^/Mg^2+^ exchanger that is expressed along the basolateral membrane.

The expression and activity of TRPM6 are upregulated by oestrogens [8]. Across species, many other aspects of kidney function and structure are known to be regulated by sex hormones as well [9–11]. The kidney mass of a female rat is approximately half that of a male rat [11]. The single-nephron glomerular filtration rate (SNGFR) in female rat kidneys is lower than in male rat kidneys, even though they have similar glomerulus population [12]. Similar to urinary output, which is not substantially different between the sexes [11], Mg^2+^ excretion has also been observed to be only ~8% higher in male rats compared with female rats [13]. Major sex differences in the abundance pattern of electrolyte transporters, channels and claudins, collectively referred to as transporters, have been reported [11,14]. Overall, in rats and mice, the female-to-male ratios for Na^+^ and water transporter abundance are ≤1 along the proximal tubule to medullary thick ascending limb, and ≥1 along the downstream segments, from cTAL through the collecting duct. The proximal pattern is partially attributed to shorter proximal tubules in females versus males in rodents [11].

Given these morphological, hemodynamic and transport capacity differences between the sexes, how does nephron segmental transport differ between the sexes to yield the reported Mg^2+^ excretion rates? Furthermore, to what extent do these differences reflect life stages that are unique to females? Specifically, while males develop to adulthood, mate and age, females may undergo serial pregnancies and lactation and then menopause—normal life cycle changes that challenge renal transporter expression, abundance and activity to maintain homeostasis. As such, the female-specific renal transport abundance pattern may give female rats the reserve transport capacity to meet the markedly altered demands of Mg^2+^ and other electrolytes in pregnancy and lactation. A better understanding of the above issue would shed insight into some of the different male and female responses to physiological, pathophysiological and pharmaceutical challenges. Towards this goal, we formulated the first set of computational models of renal epithelial transport and conducted simulations to predict the transport of Mg^2+^, as well as other electrolytes and water, along the superficial nephron of the male and female rat kidney. We formulated separate models for a male rat and a female rat and applied those models to investigate the functional implications of sex differences in kidney structure and transport.

## 2. Methods

We have previously published a series of epithelial cell-based computational models of transporter-mediated solute and water transport along the nephron of a rat kidney [15–18]. The focus of those studies was on the renal handling of Na^+^, K^+^, glucose and water in physiological and pathophysiological conditions [16,17], the associated oxygen consumption [15,16], and the functional impacts of sex differences in renal morphology, hemodynamics and transporter pattern [18]. Those models were recently extended to simulate Ca^2+^ transport along the rat kidney [19–22]. In this study, we extend the models to investigate Mg^2+^ transport along a superficial nephron in the kidney of a male rat and a female rat.

The superficial nephron model includes the proximal tubule, short descending limb, thick ascending limb, DCT, connecting tubule and collecting duct segments. Each nephron segment is represented as a tubule lined by a layer of epithelial cells. The model tracks the transport of the following 17 solutes: Na^+^, K^+^, Cl^−^, HCO_3_^−^, H_2_CO_3_, CO_2_, NH_3_, NH_4_^+^, HPO_4_^2−^, H_2_PO_4_^−^, H^+^, HCO_2_^−^, H_2_CO_2_, urea, glucose, Ca^2+^ and Mg^2+^. The segment and cell types determine the type and abundance of transporters found on the apical and basolateral membranes of the cell. Solutes and water may be transported across the epithelium either by moving across the apical and basolateral membranes in the transcellular pathway, mediated by specialized membrane transporters or channels, or via the paracellular pathway between neighbouring cells. The model is defined by a large system of coupled differential and algebraic equations that describe mass conservation and determine transmembrane and paracellular fluxes [23]. The model predicts luminal fluid flow, hydrostatic pressure, membrane potential, luminal and cytosolic solute concentrations, transcellular and paracellular fluxes, urine volume, and urinary excretion rates of model solutes.

**Figure 1.**
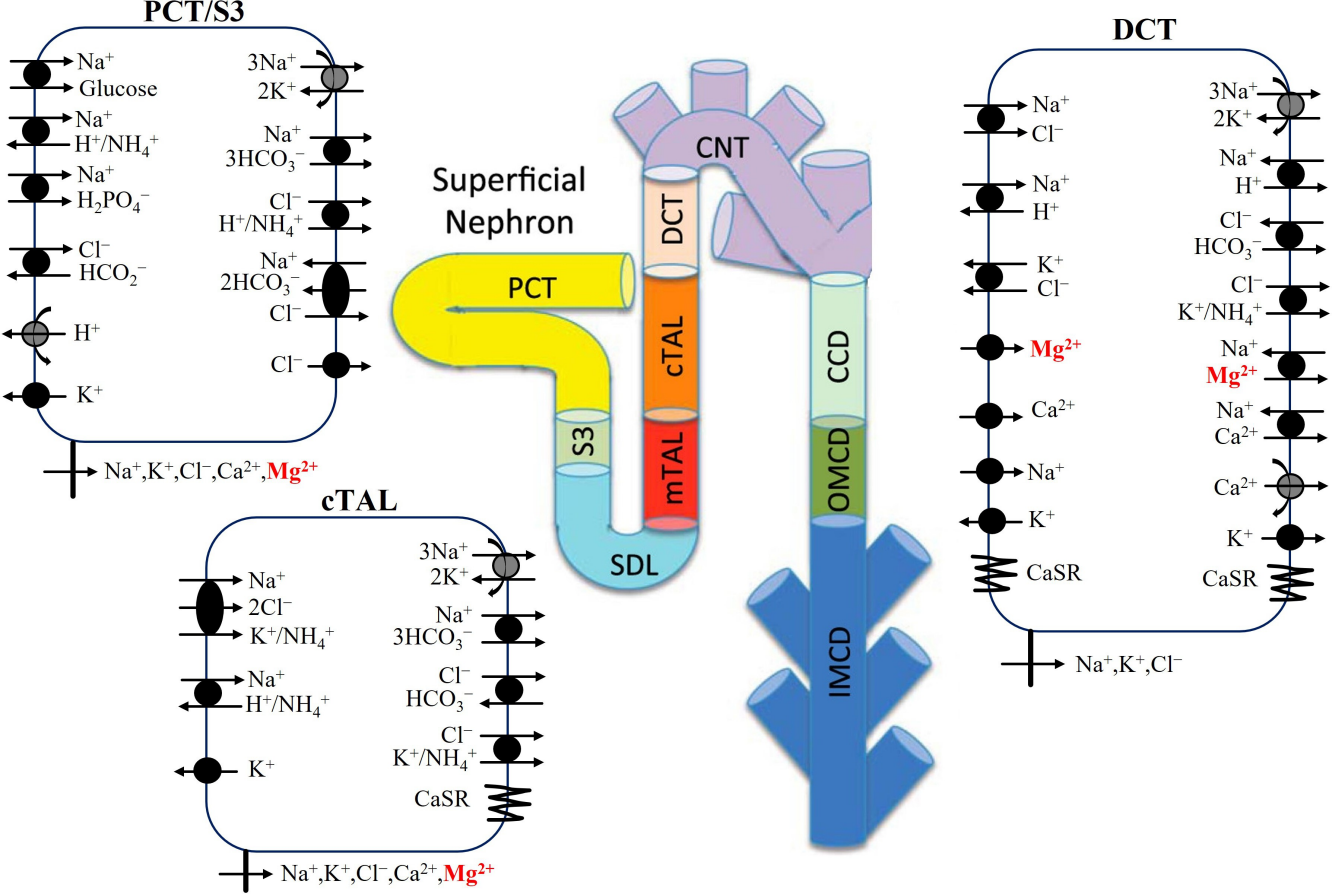
Model diagram of epithelial transport of Mg^2+^ and selected electrolytes along the superficial nephron. Mg^2+^ transport occurs along the proximal tubule (PCT and S3), cTAL and DCT. Only the major Na^+^, K^+^, Cl^−^, Ca^2+^ and Mg^2+^ transporters are shown. PCT, proximal convoluted tubule; SDL, short descending limb; mTAL, medullary thick ascending limb; CNT, connecting tubule; CCD, cortical collecting duct; OMCD, outer-medullary collecting duct; IMCD, inner-medullary collecting duct. Nephron image adapted from [15].

The details of how Mg^2+^ transport is modelled along the proximal tubule, cTAL and DCT are given in the following sections. A schematic diagram of the model nephron and the three epithelial cell types that mediate Mg^2+^ transport is given in figure 1. Male and female rat models differ in parameters describing SNGFR, tubular dimensions, membrane transporter and channel activities, and paracellular permeabilities. Model parameters that describe Mg^2+^ transport are given in table 1. Additional model parameters can be found in [22].

**Table 1.** Mg^2+^-specific parameters for all the segments along the superficial nephron. Values marked (*) are adjusted and those marked (**) are the same for both male and female models. PT, proximal tubule; TAL, thick ascending limb; DCT, distal convoluted tubule; CNT, connecting tubule; CD, collecting duct; OMCD, outer-medullary collecting duct; IMCD, inner-medullary collecting duct; NCC, Na^+^–Cl^−^ cotransporter.

parameter	value		details
	male	female	
**PT**			
tight junction permeability to Mg^2+^ at the lumen–LIS interface ( $P_{\text{Mg}}^{\text{PT,LI}}$ )	1.1 × 10^−5^cm/s**	1.1 × 10^−5^ cm/s [24]	single nephron microperfusion experiment on adult female Wistar rats
reflection coefficient of tight junction to Mg^2+^	0.89*	0.89**	assumed to be the same as the reflection coefficient of tight junction to Ca^2+^
**cTAL**			
tight junction permeability at the lumen–LIS interface in the absence of Mg^2+^ ( $P_{\text{Mg}}^{\text{TAL,LI*}}$ )	38 × 10^−5^ cm/s*	56 × 10^−5^ cm/s*	estimated (refer to §2.5)
maximum half concentration of Ca^2+^ ( ${EC}_{50,\text{Ca}}$ )	1.25 mM [25]	1.25**	*in vitro* study on bovine parathyroid cells
maximum half concentration of Mg^2+^ ( ${EC}_{50,\text{Mg}}$ )	2.5 mM [25]	2.5**	*in vitro* study on bovine parathyroid cells
Hill function coefficient, *n*	4 [25]	4**	*in vitro* study on bovine parathyroid cells
inhibitory coefficient of Ca^2+^ on tight junction permeability ( $\alpha_{\text{P,Ca}}$ )	−4/7 [26]	−4/7**	*in vitro* microperfusion experiment on male Sprague Dawley rats
inhibitory coefficient of Mg^2+^ on tight junction permeability ( $\alpha_{\text{P,Mg}}$ )	−0.34*	−0.34**	estimated (refer to §2.4)
inhibitory coefficient of Ca^2+^ on NKCC_2_ activity ( $\alpha_{\text{NKCC2,Ca}}$ )	−0.4 [27]	−0.4**	*in vivo* study on mice (sex not specified)
inhibitory coefficient of Mg^2+^ on NKCC_2_ activity ( $\alpha_{\text{NKCC2,Mg}}$ )	−0.24*	−0.24**	estimated (refer to §2.4)
inhibitory coefficient of Ca^2+^ on ROMK activity ( $\alpha_{\text{ROMK,Ca}}$ )	−0.8 [28,29]	−0.8**	*in vitro* study on rat (sex not specified)
inhibitory coefficient of Mg^2+^ on ROMK activity ( $\alpha_{\text{ROMK,Mg}}$ )	−0.48*	−0.48**	estimated (refer to §2.4)
**DCT**			
TRPM6 channel density ( $N_{TRPM6}$ )	22.5 × 10^4^1/cm^2^*	45 × 10^4^ 1/cm^2^*	estimated (refer to §2.5)
single channel conductance of TRPM6 at pH 7.4 ( $g_{\text{pH7.4}}$ )	83.6 pS [30]	83.6 pS**	*in vitro* study using CHOK1 cells
luminal pH for half-maximal conductance of TRPM6 ( ${\text{pH}}_{1/2}$ )	4.3 [30]	4.3**	*in vitro* study using CHOK1 cells
maximum Mg^2+^ flux through Na^+^/Mg^2+^ exchanger ( $J_{\text{Mg}}^{\text{NaMgX,max}}$ )	8.6 × 10^−9^ mmol/cm^2^/s*	9 × 10^−9^ mmol/cm^2^/s*	estimated (refer to §2.5)
intracellular Mg^2+^ half-saturation constant ( $K_{M,\text{Mg}C}$ )	3.59 M [31]	3.59 M**	*in vitro* study using rat erythrocyte cells
extracellular Mg^2+^ half-saturation constant ( $K_{M,\text{Mg}S}$ )	1.3 mM [31]	1.3 mM**	*in vitro* study using rat erythrocyte cells
intracellular Na^+^ half-saturation constant ( $K_{M,\text{Na}C}$ )	12.29 mM [31]	12.29 mM**	*in vitro* study using rat erythrocyte cells
extracellular Na^+^ half-saturation constant ( $K_{M,\text{Na}S}$ )	87.5 mM [31]	87.5 mM**	*in vitro* study using rat erythrocyte cells
excitatory coefficient of Ca^2+^ on NCC activity ( $\alpha_{\text{NCC,Ca}}$ )	0.5 [32]	0.5**	*in vivo* study on C57BL/6 male mice
excitatory coefficient of Mg^2+^ on NCC activity ( $\alpha_{\text{NCC,Mg}}$ )	0.3*	0.3**	estimated (refer to §2.4)
**CD**			
Ca^2+^ promoting coefficient for apical HATPase activity of type A OMCD cells ( $\alpha_{\text{HATP,Ca}}$ )	2 [26]	2**	*in vivo* study on mice
Mg^2+^ promoting coefficient for apical HATPase activity of type A OMCD cells ( $\alpha_{\text{HATP,Mg}}$ )	1.2*	1.2**	estimated (refer to §2.4)
Ca^2+^ inhibitory coefficient for apical water permeability of IMCD cells ( $\alpha_{\text{Pf,Ca}}$ )	−3/8 [26]	−3/8**	*in vitro* study on Sprague Dawley rat cells (sex not specified)
Mg^2+^ inhibitory coefficient for apical water permeability of IMCD cells ( $\alpha_{\text{Pf,Mg}}$ )	−0.225*	−0.225**	estimated (refer to §2.4)

### 2.1. Proximal tubule

The proximal tubule reabsorbs 15–25% of the filtered Mg^2+^ load through the paracellular pathway, driven by the favourable electrochemical gradient established by Na^+^/H^+^ exchanger 3 (NHE3)-mediated Na^+^ reabsorption [5,33,34]. The paracellular electrodiffusive flux ( $J_{\text{Mg}}^{\text{PT,LI}}$ ) is given by


(2.1) $$J_{\text{Mg}}^{\text{PT,LI}}=P_{\text{Mg}}^{\text{PT,LI}}\zeta_{\text{Mg}}^{\text{LI}}\left(\frac{C_{\text{Mg}}^{\text{L}}-C_{\text{Mg}}^{\text{I}}e^{-\zeta_{\text{Mg}}^{\text{LI}}}}{1-e^{-\zeta_{\text{Mg}}^{\text{LI}}}}\right),$$

where the superscripts L and I denote lumen and lateral intercellular space (LIS), respectively; $P_{\text{Mg}}^{\text{PT,LI}}$ denotes the permeability of Mg^2+^ at the lumen and LIS interface; ζMgLI=ZMgFRTψL-ψI ; $Z_{\text{Mg}}$ is the valence of Mg^2+^ (+2); $C_{\text{Mg}}^{\text{L}}$ and $C_{\text{Mg}}^{\text{I}}$ denote Mg^2+^ concentrations in the lumen and LIS, respectively; $\psi^{L}$ and $\psi^{I}$ denote the luminal and LIS membrane potentials, respectively; RT = 2.57 J/mmol; and *F* = 96.5 C/mmol represents Faraday’s constant.

Paracellular Mg^2+^ transport is believed to be mediated by claudin-2 and claudin-12 [35]. The permeability of Mg^2+^ at the LIS has been measured in adult female rats to be ~1.1 × 10^−5^ cm/s [24]. The permeability for the basement membrane is estimated based on the permeability to Na^+^, given by a Mg^2+^-to-Na^+^ free diffusivity ratio of 7.05:13.3 [36]. The reflection coefficient of Mg^2+^ has not been measured and is assumed to be the same as Ca^2+^ (0.89), as the two divalent ions have similarly limited paracellular convective transport (solvent drag). We varied the reflection coefficient of Mg^2+^ from 0.5 to 1.0. The resulting fractional excretion of Mg^2+^ varied from 2.2% to 3% for male rats and 2.4% to 3.5% for female rats. These values are within the physiological range of 2–5% of fractional excretion of Mg^2+^. Thus, varying the reflection coefficient of Mg^2+^ does not cause a major change in Mg^2+^ excretion in either male or female rats.

### 2.2. Thick ascending limb

The cTAL reabsorbs 60–70% of the filtered Mg^2+^ via the paracellular route driven by the electrochemical gradient established by Na^+^–K^+^–Cl^−^ cotransporter 2 (NKCC2)-mediated Na^+^ transport [33,34]. Claudin-16 and claudin-19 are the main claudins that regulate Mg^2+^ permeability [7]. The medullary thick ascending limb has negligible Mg^2+^ reabsorption [37–39]. Paracellular Mg^2+^ transport in the cTAL $J_{\text{Mg}}^{\text{cTAL,LI}}$ is represented by equation (2.1) with the superscript ‘PT’ replaced by ‘cTAL’.

### 2.3. Distal convoluted tubule

As previously noted, the DCT reabsorbs 5–23% of the filtered Mg^2+^. This wide range of fractional reabsorption allows the segment to play a crucial role in fine-tuning urinary Mg^2+^ excretion, as nephron segments that are further downstream do not have the capacity for significant Mg^2+^ transport. The net Mg^2+^ reabsorption in the DCT is unidirectional as no secretion of Mg^2+^ has been reported [40]. Since the DCT has a lumen-negative transepithelial potential and high epithelial resistance, Mg^2+^ reabsorption in this segment is active and transcellular and is mediated by the TRPM6 channels, expressed on the apical membranes [41]. TRPM6 is inhibited by intracellular Mg^2+^ concentration ( $C_{\text{Mg}}^{\text{C}}$ ) and the effect is given by


(2.2) $$f_{\text{Mg}}=\frac{1}{1+{\left(\frac{C_{\text{Mg}}^{\text{C}}}{0.51}\right)}^{2}}.$$

Lowering extracellular pH increases TRPM6 inward currents [30]. This effect is modelled as


(2.3) $$f_{\text{pH}}=g_{\text{pH7.4}}\left(2-\frac{1}{1+\frac{7.4-{\text{pH}}^{\text{L}}}{7.4-{\text{pH}}_{1/2}}}\right),$$

where $g_{\text{pH7.4}}$ denotes the single channel conductance of TRPM6 at pH 7.4, ${\text{pH}}^{\text{L}}$ denotes the luminal fluid pH and ${\text{pH}}_{1/2}$ denotes the luminal fluid pH for half-maximal conductance.

The Mg^2+^ flux through TRPM6 also depends on the luminal Mg^2+^ concentration [42] and is modelled as


(2.4) $$f_{\text{concL}}=\frac{1}{1+\frac{0.8}{C_{\text{Mg}}^{\text{L}}}}.$$

Hence, the flux of Mg^2+^ through TRPM6 is given by


(2.5) $$J_{\text{Mg}}^{\text{TRPM6}}=N_{\text{TRPM6}}\times f_{\text{Mg}}\times f_{\text{pH}}\times \frac{\Delta \psi^{LC}-E_{\text{Mg}}^{LC}}{2F}\times f_{\text{concL}},$$

where $N_{\text{TRPM6}}$ denotes the TRPM6 channel density, $\Delta \psi^{LC}$ denotes the potential difference across the apical membrane and $E_{\text{Mg}}^{LC}$ denotes the Nernst potential of Mg^2+^:


(2.6) $$E_{\text{Mg}}^{LC}=\frac{RT}{2F}\times \ln\left(\frac{C_{\text{Mg}}^{\text{C}}}{C_{\text{Mg}}^{\text{L}}}\right).$$

Mg^2+^ efflux through the basolateral membrane is not well characterized. Mg^2+^ efflux likely requires an anti-porter or ATPase, since no chemical gradient exists for Mg^2+^ (with both intracellular and extracellular Mg^2+^ concentrations being in the range of 0.4–1 mM), while the potential gradient favours Mg^2+^ influx. A Na^+^/Mg^2+^ exchanger has been demonstrated as the mechanism for Mg^2+^ efflux in multiple cell types [41,43]. The molecular identity of the Na^+^/Mg^2+^ exchanger remains to be definitively identified, but the most promising candidate is the solute carrier family 41 member 1 (*SLC41A1*), which has been shown to facilitate Mg^2+^ efflux [41]. Na^+^/Mg^2+^ exchanger activity is regulated by the following [43]:

(i) extracellular Na^+^, $f_{\text{Na}S}=\frac{{\left(c_{\text{Na}}^{\text{S}}\right)}^{2}}{\left({\left(c_{\text{Na}}^{\text{S}}\right)}^{2}+{\left(K_{M,\text{Na}S}\right)}^{2}\right)},$

(ii) intracellular Na^+^, $f_{\text{Na}C}=\frac{K_{M,\text{Na}C}}{\left(c_{\text{Na}}^{\text{C}}+K_{M,\text{Na}C}\right)},$

(iii) extracellular Mg^2+^, $f_{\text{Mg}S}=\frac{K_{M,\text{Mg}S}}{\left(c_{\text{Mg}}^{\text{S}}+K_{M,\text{Mg}S}\right)},$ and

(iv) intracellular Mg^2+^, $f_{\text{Mg}C}=\frac{{\left(c_{\text{Mg}}^{\text{C}}\right)}^{2}}{\left({\left(c_{\text{Mg}}^{\text{C}}\right)}^{2}+{\left(K_{M,\text{Mg}C}\right)}^{2}\right)},$

where $c_{\text{Na}}^{\text{S}}$ and $c_{\text{Mg}}^{\text{S}}$ denote interstitial fluid Na^+^ and Mg^2+^ concentrations, respectively, and $K_{M,\text{Na}S}$ , $K_{M,\text{Na}C}$ , $K_{M,\text{Mg}S}$ and $K_{M,\text{Mg}C}$ denote the Michaelis–Menten constants. Hence, the Na^+^/Mg^2+^ exchanger-mediated Mg^2+^ flux is formulated as


(2.7) $$J_{\text{Mg}}^{\text{NaMgX}}=J_{\text{Mg}}^{\text{NaMgX,max}}\times f_{\text{Na}S}\times f_{\text{Na}C}\times f_{\text{Mg}S}\times f_{\text{Mg}C},$$

where $J_{\text{Mg}}^{\text{NaMgX,max}}$ denotes the maximum Mg^2+^ flux through Na^+^/Mg^2+^ exchanger. The estimation of the Mg^2+^ transporter parameters, $N_{\text{TRPM6}}$ and $J_{\text{Mg}}^{\text{NaMgX,max}}$ , is explained in §2.5.

### 2.4. Calcium-sensing receptor

The calcium-sensing receptor (CaSR) plays an important role in Ca^2+^ and Mg^2+^ homeostasis by controlling parathyroid hormone (PTH) secretion from the parathyroid gland as well as regulating Ca^2+^ and Mg^2+^ reabsorption in the kidneys. CaSR is ubiquitously expressed in the kidney along both the apical (proximal convolulted tubule, DCT and inner-medullary collecting duct) and basolateral membranes (thick ascending limb, DCT, cortical and outer-medullary collecting duct), with its highest expression being at the basolateral membrane of the cTAL [44]. Ca^2+^ is the primary ligand for activating CaSR. At equimolar concentrations, Mg^2+^ is 1/2 to 2/3 as potent as Ca^2+^ in activating CaSR [31,45]. We model the effect of CaSR on a given parameter *v* (*v* may denote paracellular permeability, NKCC2 activity, renal outer-medullary potassium channel (ROMK) activity or Na^+^–Cl^−^ cotransporter (NCC) activity; see below) with the following function:


(2.8) $$v=\;v^{*}\left(1+\alpha_{v,\text{Ca}}\left(\frac{{\left(C_{\text{Ca}}^{i}\right)}^{n}}{{\left(C_{\text{Ca}}^{i}\right)}^{n}+{\left(EC_{50,\text{Ca}}\right)}^{n}}\right)\right)\left(1+\alpha_{v,\text{Mg}}\left(\frac{{\left(C_{\text{Mg}}^{i}\right)}^{n}}{{\left(C_{\text{Mg}}^{i}\right)}^{n}+{\left(EC_{50,\text{Mg}}\right)}^{n}}\right)\right),$$

where $v^{*}$ is the value of *v* in the absence of the effect of CaSR, $c_{\text{Ca}}^{i}$ and $c_{\text{Mg}}^{i}$ denote the concentration of Ca^2+^ and Mg^2+^, respectively, in the luminal (*i* = L) or interstitial (*i* = S) fluid, ${EC}_{50,\text{Ca}}$ = 1.25 mM and ${EC}_{50,\text{Mg}}$ = 2.5 mM represent the half-maximal concentrations for Ca^2+^ and Mg^2+^ [25], respectively, and *n* is the Hill coefficient, which is set to 4 because of the steep relationship between extracellular Ca^2+^ and Mg^2+^ concentrations and PTH release [25]. The parameters $\alpha_{v,\text{Ca}}$ and $\alpha_{v,\text{Mg}}$ are negative if CaSR has an inhibitory effect on *v* and positive otherwise. Since the effect of Mg^2+^ on CaSR activation is ~50–66% of that of Ca^2+^, we set $\alpha_{v,\text{Mg}}$ = 0.6 $\alpha_{v,\text{Ca}}$ . We set the value for $\alpha_{v,\text{Ca}}$ and $\alpha_{v,\text{Mg}}$ for each of the following parameters as follows:

— *Paracellular permeability in the thick ascending limb*: Inhibition of CaSR increases paracellular permeability of Ca^2+^ by 41% along the thick ascending limb without any changes in Na^+^ and Cl^−^ fluxes [46]. So, we set $\alpha_{\text{P,Ca}}$ to −4/7 [26] and $\alpha_{\text{P,Mg}}$ = 0.6 × (−4/7) = −0.34. The cTAL permeability to Mg^2+^ in the absence of the effect of CaSR ( $P_{\text{Mg}}^{\text{c}\text{TAL,LI*}}$ ) is taken as 38 × 10^−5^ cm/s in male rats and 56 × 10^−5^ cm/s in female rats, so that under basal conditions, $P_{\text{Mg}}^{\text{c}\text{TAL,LI}}$ is equal to 27.2 × 10^−5^ cm/s in male rats and 40 × 10^−5^ cm/s in female rats. These sex differences are explained in §2.5.
— *NKCC2 activity in the thick ascending limb*: NKCC2 phosphorylation decreased by ~40% in mice treated with calcimimetic [32]. In another study, targeted deletion of CaSR in the kidney of mice resulted in a significant increase in NKCC2 phosphorylation [27]. Hence, we set $\alpha_{\text{NKCC2,Ca}}$ to −0.4 and $\alpha_{\text{NKCC2,Mg}}$ = 0.6 × (−0.4) = −0.24.
— *ROMK activity in the thick ascending limb*: Increasing extracellular Ca^2+^ concentration from 1.1 to 5 mM decreased ROMK channel activity in the thick ascending limb by ~84% [28,29]. Thus, we set $\alpha_{\text{ROMK,Ca}}$ to −0.8 and $\alpha_{\text{ROMK,Mg}}$ = 0.6 × (−0.8) = −0.48.
— *NCC activity in the DCT*: NCC phosphorylation increased by ~50% in mice treated with calcimimetic [32]. Hence, we set $\alpha_{\text{NCC,Ca}}$ to 0.5 and $\alpha_{\text{NCC,Mg}}$ = 0.6 × 0.5 = 0.3.
— *H* ^*+*^ *-ATPase flux in outer-medullary collecting duct type A cells*: Increasing luminal Ca^2+^ concentration from 0.1 to 5 mM increased the rate of intracellular pH recovery in OMCD; that is, the proton flux across H^+^-ATPase in outer-medullary collecting duct type A cells increased. Hence, $\alpha_{\text{HATP,Ca}}$ is set to 2 [26] and $\alpha_{\text{HATP,Mg}}$ = 0.6 × 2 = 1.2.
— *Water permeability in the inner-medullary collecting duct*: Increasing luminal Ca^2+^ concentration from 1 to 5 mM decreased the water permeability of the apical membrane in the inner-medullary collecting duct of rat by 30% [47]. Hence, we set $\alpha_{\text{Pf,Ca}}$ to −3/8 [26] and $\alpha_{\text{Pf,Mg}}$ = 0.6 × (−3/8) = −0.225.

### 2.5. Sex differences in Mg^2+^ transport parameters

Along the proximal tubule, Mg^2+^ paracellular permeability is assumed to be the same in both sexes. Sex differences in the transport properties for Na^+^ and other electrolytes yield a higher fractional reabsorption of Na^+^ and water in male rats relative to females. Consequently, the luminal Mg^2+^ concentration rises more in males. This does not manifest in any notable differences in proximal tubule Mg^2+^ reabsorption due to its low Mg^2+^ permeability. However, Mg^2+^ transport rate is several times higher along the cTAL due to its higher Mg^2+^ paracellular permeability. The absolute reabsorption rate of Mg^2+^ along this segment is ~7–11 pmol/min for male and female rats (the experiment did not report sex-specific values) [40]. For the male model, we estimate the paracellular permeability of Mg^2+^, $P_{\text{Mg}}^{\text{c}\text{TAL,LI}}$ , to be 27.2 × 10^−5^ cm/s so that the absolute reabsorption rate of Mg^2+^ along this segment is ~10 pmol/min, which corresponds to ~69% fractional Mg^2+^ reabsorption. To maintain a similar fractional Mg^2+^ reabsorption along the cTAL in both sexes despite the higher driving force (luminal Mg^2+^ concentration) in males, we assume that the Mg^2+^ paracellular permeability is higher in females (40 × 10^−5^ cm/s). The absolute reabsorption rate of Mg^2+^ along the DCT lies in the range of 1–1.7 pmol/min for male and female rats (the experiment did not report sex-specific values) [40]. Hence, for the male rat model, the parameters $N_{\text{TRPM6}}$ and $J_{\text{Mg}}^{\text{NaMgX,max}}$ are estimated so that the absolute reabsorption rate of Mg^2+^ along this segment is ~1 pmol/min. TRPM6 expression was found to be twofold higher in non-pregnant female mice than in male mice [48]. Hence, we assume the female $N_{\text{TRPM6}}$ value to be double the male $N_{\text{TRPM6}}$ value. Finally, Mg^2+^ excretion has been observed to be ~8% higher in male rats compared with female rats [13].

### 2.6. Simulating the effect of loop diuretics

Loop diuretics inhibit NKCC2, which is expressed on the apical membrane of the thick ascending limb. We simulated the effect of acute administration of loop diuretics by inhibiting NKCC2 activity by 70%. We assumed that the NKCC2 inhibitor was administrated for long enough to significantly impair the kidney’s ability to generate an axial osmolality gradient. The cortical interstitial concentrations were assumed to remain unchanged. Since the concentrating mechanism of the outer medulla is significantly impaired following complete NKCC2 inhibition, the interstitial concentration of Mg^2+^ at the outer–inner medullary boundary is lowered to 0.77 mM (from a baseline value of 0.96 mM). At the papillary tip, the interstitial concentration of Mg^2+^ is reduced from 1.54 to 1.01 mM. For changes in the interstitial concentrations of Na^+^, K^+^, Cl^−^, urea and Ca^2+^, refer to [16,22].

### 2.7. Simulating the effect of thiazide diuretics

Thiazide diuretics inhibit NCC, which is expressed along the apical membrane of the DCT. We simulated the effect of acute administration of thiazide diuretics by inhibiting NCC activity by 70%. In the NCC inhibition simulations, baseline interstitial concentration profiles were used.

### 2.8. Simulating the effect of K-sparing diuretics

K-sparing diuretics, such as amiloride, block Na^+^ uptake through epithelial Na^+^ channels (ENaC), expressed on the apical membrane of the late DCT as well as along the full length of the connecting tubule and collecting ducts. K-sparing diuretics are weak diuretics often used in combination with others to lower blood pressure. By inhibiting ENaC, these diuretics hyperpolarize the luminal membrane potential and increase K^+^, Ca^2+^ and Mg^2+^ uptake [49–52]. In our model, we simulated the effect of K-sparing diuretics by reducing ENaC activity by 70%.

## 3. Results

### 3.1. Baseline results

**Figure 2.**
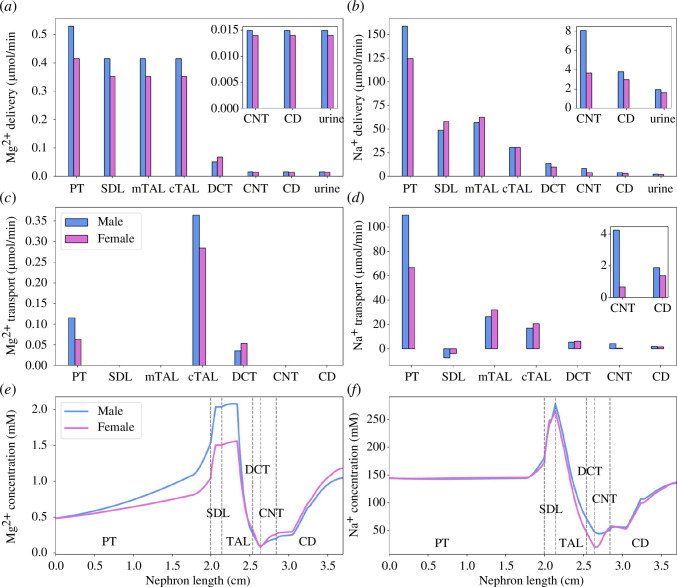
Baseline model predictions. (*a, b*) Delivery of Mg^2+^ and Na^+^ to key nephron segments in male and female rats, given per kidney. (*c, d*) Mg^2+^ and Na^+^ transepithelial transport along key nephron segments in male and female rats, given per kidney. (*e, f*) Luminal Mg^2+^ and Na^+^ concentrations along key nephron segments in male and female rats. PT, proximal tubule; SDL, short descending limb; mTAL/cTAL, medullary/cortical thick ascending limb; DCT, distal convoluted tubule; CNT, connecting tubule; CD, collecting duct.

Figure 2 shows the segmental delivery, transport and luminal fluid concentration of Mg^2+^ and Na^+^ in male and female rats. Results for other electrolytes and water can be found in [18]. The addition of Mg^2+^ has negligible impacts on the renal transport of other major electrolytes and water, because luminal Mg^2+^ flow is orders of magnitude smaller. Filtered Mg^2+^ load is 25% higher in males due to their higher SNGFR [12,53]. Model simulations predict that fractional reabsorption of Mg^2+^ along the proximal tubule is higher in males than females (22% versus 16%), due to the more favourable electrochemical gradient generated by the higher NHE3-mediated Na^+^ transport rate in males [14]. Since Mg^2+^ reabsorption is low along the proximal tubule, Mg^2+^ concentration increases gradually along this segment. Micropuncture experiments in male rats showed that the tubular fluid-to-ultrafiltrate Mg^2+^ concentration ratio at the start of the loop of Henle was 3.24 ± 0.12 [4]. Our male model predicted this ratio to be 3.17, which is in line with the experimental observations. Single nephron microperfusion experiments with female rats showed that the luminal Mg^2+^ concentration in the late proximal tubule was 0.70 ± 0.16 mM [54]. Our female rat model predicted the luminal Mg^2+^ concentrations in the late proximal tubule to be 0.95 mM. The majority of the overall Mg^2+^ transport occurs downstream along the cTAL (69% of the filtered load in both males and females), where the lumen-positive membrane potential drives Mg^2+^ reabsorption via the paracellular pathway. Micropuncture studies in male rats found the tubular fluid-to-ultrafiltrate Mg^2+^ concentration ratio in the early distal tubule to be 0.80 ± 0.06 [4]. Our male model predicted this ratio to be 0.67. Microperfusion experiments in female rats showed that the luminal Mg^2+^ concentration in the early distal tubule was 0.37 ± 0.27 mM [54] and our female rat model predicted this value to be 0.32 mM. The final nephron segment that transports Mg^2+^ is the DCT, where the higher TRPM6 expression in females yields a higher Mg^2+^ reabsorption rate (6.6% and 12% of the filtered Mg^2+^ in males and females, respectively). Urinary Mg^2+^ excretion is predicted to be ~8% higher in males compared with females, consistent with experimental data, partially validating the baseline models [13]. However, given the 25% lower filtered load in females, fractional Mg^2+^ excretion is higher in females (3.3%) than males (2.8%) [13].

### 3.2. Effect of varying proximal tubule and thick ascending limb Mg^2+^ transport

**Figure 3.**
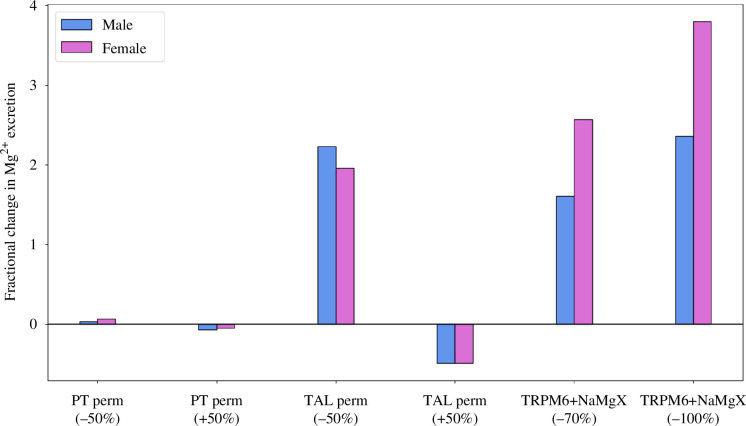
Changes in Mg^2+^ excretion by varying Mg^2+^ specific parameters. Male and female rat Mg^2+^ excretions are normalized by their respective baseline values. ‘PT perm (±50%)’, proximal tubule permeability to Mg^2+^ increased or decreased by 50%; ‘TAL perm (±50%)’, thick ascending limb permeability to Mg^2+^ increased or decreased by 50%; ‘TRPM6+NaMgX (–70%)’ and ‘TRPM6+NaMgX (–100%)’, transient receptor potential melastatin 6 and Na^+^/Mg^2+^ exchanger activity co-inhibited by 70% and 100%, respectively.

We first vary proximal tubule Mg^2+^ paracellular permeability by ±50%. This generates almost 20% changes in proximal tubular Mg^2+^ transport, which are then offset by compensatory transport along the cTAL, driven by the higher luminal Mg^2+^ concentration, resulting in essentially no change in DCT transport and only small (<6%) changes in urinary Mg^2+^ excretion rates for both male and female rats (figure 3). The effects on other electrolytes and water are minimal.

When Mg^2+^ paracellular permeability is increased by 50% along the cTAL, the models predict small relative increases in segmental transport (2% in males and 3% in females), which are partially compensated by opposing changes (decreases) in DCT transport. Because only 2–5% of the filtered Mg^2+^ is typically excreted in urine, Mg^2+^ excretion decreases by ~50% in both sexes (figure 3). Decreasing the Mg^2+^ paracellular permeability along the cTAL by 50% has a larger effect, with a ~10% decrease in segmental transport in males and females. With partial compensation by the DCT, Mg^2+^ urinary excretion increases by 220% in males and 196% in females (figure 3). Further model validation can be done by comparing these predictions with knockout measurements. Mutations in claudin-16 and claudin-19, which determine the paracellular permeability of Mg^2+^ in the thick ascending limb, are associated with increased Mg^2+^ excretion [55,56]. Since this segment is responsible for the majority of Mg^2+^ reabsorption and since the downstream distal segment has limited Mg^2+^ reabsorption capacity, a 50% decrease in permeability is predicted to significantly increase Mg^2+^ excretion, as is observed from experimental data of claudin-16 and claudin-19 knockout rodents [55,56]. The effect on urinary excretion is smaller in females due to their higher distal transport capacity. But even in this case, the effects on other electrolytes are minimal.

### 3.3. Effect of TRPM6 and Na^+^/Mg^2+^ exchanger co-inhibition

The DCT reabsorbs Mg^2+^ through TRPM6 on the apical membrane and Na^+^/Mg^2+^ exchanger on the basolateral membrane. We conduct simulations where we inhibit TRPM6 and Na^+^/Mg^2+^ exchanger simultaneously by 70% and then completely. Results for these two cases suggest that distal Mg^2+^ uptake scales linearly with inhibition. Without compensatory transport in any downstream segments, inhibition of DCT transport has marked effects on Mg^2+^ excretion. With 70% co-inhibition, Mg^2+^ excretion increases by 160% and 250% in male and female rats, respectively; with full inhibition, excretion rates increase by 240% and 380% in males and females, respectively. This is in line with the observation that mutations in TRPM6 lead to high Mg^2+^ wasting and hypomagnesemia [57,58]. In contrast to the previous case where thick ascending limb Mg^2+^ permeability is varied, here the effect on urinary excretion is larger in females because their DCT is responsible for transporting a larger fraction of the filtered Mg^2+^. Again, there is little impact on other electrolytes.

### 3.4. Effects of sex differences in transporter patterns

The model represents the sexual dimorphism in expression patterns not only for Mg^2+^ but also for other electrolytes. Specifically, female rats have lower proximal tubule NHE3 activity and higher NKCC2, NCC and distal segmental Na^+^–K^+^–ATPase activities (these transporters are coupled to Mg^2+^ transport) [14]. To what extent does each of these individual factors, and the sex differences in Mg^2+^ transporter properties, contribute to the sex differences in Mg^2+^ excretion?

**Figure 4.**
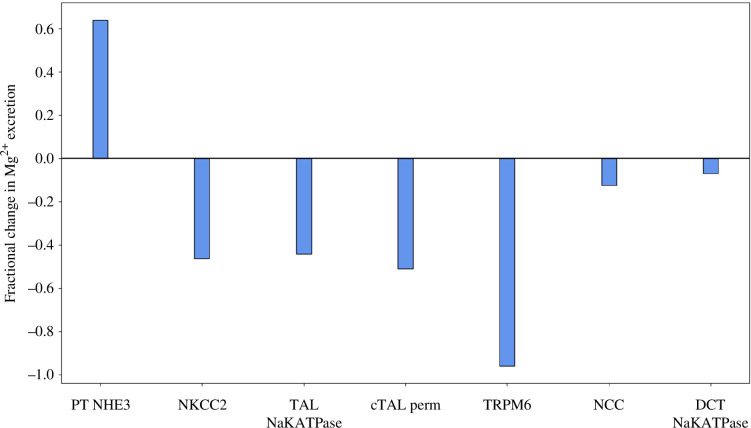
Effect of individual sex-specific model parameters. Changes in Mg^2+^ excretion relative to baseline male values when individual parameters in the male rat model are set to female rat values. PT NHE_3_, proximal tubule Na^+^–H^+^ exchanger; NKCC2, Na^+^–K^+^–2Cl^−^ cotransporter; TAL NaKATPase, thick ascending limb Na^+^–K^+^–ATPase; cTAL perm, cortical thick ascending limb permeability to Mg^2+^; TRPM6, transient receptor potential melastatin 6; NCC, Na^+^–Cl^−^ cotransporter; DCT NaKATPase, distal convoluted tubule Na^+^–K^+^–ATPase.

To answer that question, we conduct simulations to analyse the extent to which each sex difference in transporter activity contributes to the differences in Mg^2+^ excretion. To that end, we vary each sex-specific parameter individually in the male rat nephron model and compute the fractional change in Mg^2+^ excretion. For example, the ‘PT NHE3’ case consists of male rat parameters, except for proximal tubule NHE3 activity, which is set to the female value. Simulation results are summarized in figure 4.

When NHE3 activity in the male model is lowered by 17% to match the female value, the favourable electrochemical gradient that drives Mg^2+^ is attenuated, reducing proximal tubule fractional Mg^2+^ reabsorption from the baseline by 22% to 20%. Compensatory transport by downstream segments is limited, unlike the case when proximal tubule Mg^2+^ permeability was reduced (see above). This discrepancy can be attributed to the effect of NHE3 inhibition on water transport. Here, the reduction in NHE3 activity lowers the reabsorption not only of Mg^2+^ but also of Na^+^, K^+^, Cl^−^ and water. Consequently, the luminal Mg^2+^ concentration does not differ substantially from the base case (unlike when proximal tubule Mg^2+^ permeability was reduced), resulting in limited compensatory transport along the thick ascending limb and DCT, and a marked 64% increase in Mg^2+^ excretion.

Along the cTAL, NKCC2 activity, Na^+^–K^+^–ATPase activity and paracellular Mg^2+^ permeability are higher in females, by 100%, 100% and 47%, respectively, compared with males [14]. When each of these parameters are set to the female value, cTAL fractional Mg^2+^ reabsorption increases, resulting in a decrease in Mg^2+^ excretion (by 46%, 44% and 51%, respectively). Similarly, increasing TRPM6 channel density by 100% to match the female value increases Mg^2+^ reabsorption along the DCT and decreases Mg^2+^ excretion by 96% to almost nil. Also, along the DCT, NCC and Na^+^–K^+^–ATPase activities are higher in females [14]. Setting each of these parameters to the female value decreases Mg^2+^ excretion, albeit the reductions are lower than the other changes (refer to figure 4). The higher (almost doubled) TRPM6 channel density in females has the largest impact on Mg^2+^ excretion because it directly affects Mg^2+^ transport and also because there is no downstream segment to compensate for Mg^2+^ transport. Among transporters that do not immediately mediate Mg^2+^ excretion, the lower proximal tubule NHE3 activity in females has the largest impact on Mg^2+^ excretion, even though less Mg^2+^ is reabsorbed along the proximal tubule than along the cTAL, indicating a stronger coupling between Na^+^ and Mg^2+^ transport along the proximal tubule.

### 3.5. Effect of diuretics

To validate the model, we simulate the administration of three classes of diuretics (loop diuretics, thiazide diuretics and K-sparing diuretics) and compute model predictions with observations in rodents.

#### 3.5.1. Loop diuretics

**Figure 5.**
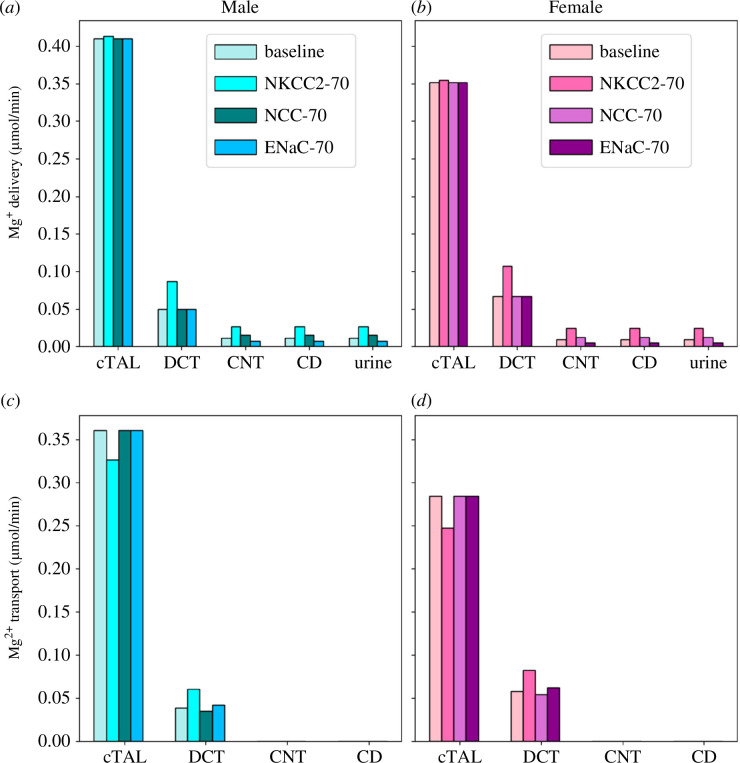
(*a*–*d*) Delivery and transport of Mg^2+^ along key nephron segments in male and female rats under normal condition and 70% inhibition of NKCC2, NCC and ENaC. The values are given per kidney. Notations are analogous to figure 2.

Administration of furosemide to male rats increased Mg^2+^ excretion to 240% of the control excretion value [59]. The predicted Mg^2+^ transport along the cTAL and DCT and urinary Mg^2+^ excretion following NKCC2 inhibition in male and female rats are shown in figure 5. NKCC2 inhibition decreased fractional Mg^2+^ reabsorption along male and female cTALs to 57% and 52%, respectively, from the baseline fractional reabsorption of 69% (figure 5). Females have a higher reduction in Mg^2+^ reabsorption because they have higher NKCC2 activity. In male rats, this 12% decrease in Mg^2+^ reabsorption along the cTAL should ideally increase Mg^2+^ excretion to ~430% of the control value. However, according to the experimental study [59], Mg^2+^ excretion increases to 240% of the control value following furosemide treatment. This indicates that there must be a compensatory increase in Mg^2+^ reabsorption along the DCT. Indeed, TRPM6 mRNA expression was found to be increased by 30% in male mice undergoing furosemide treatment [60]. Our male model simulations indicated that TRPM6 activity must be increased by 68% to account for the experimental increase in Mg^2+^ excretion. Due to lack of data for female rodents, we assumed the same percentage increase (68%) in TRPM6 for female rats. With a 68% increase in TRPM6 activity, the male and female model simulations predicted fractional Mg^2+^ reabsorptions along the DCT to increase to 12% and 20%, respectively (figure 5). Finally, the predicted Mg^2+^ excretions are 241% and 270% of the baseline male and female excretion values, respectively (figure 5). Furosemide has been found to cause stronger diuretic, natriuretic and kaliuretic responses in female rats [61]. Since Mg^2+^ transport is strongly coupled to Na^+^ uptake through NKCC2, it can be inferred that the stronger natriuretic response in female rats on furosemide treatment would lead to a higher increase in Mg^2+^ excretion compared with male rats.

#### 3.5.2. Thiazide diuretics

Administration of bendrofluazide, a thiazide diuretic, did not cause any significant change in Mg^2+^ excretion in male rats [62]. In agreement with the experimental data, the predicted male and female fractional Mg^2+^ excretions after NCC inhibition increased to 3.1% and 3.7%, respectively, from the baseline values of 2.8% and 3.3% (figure 5). In addition, we also performed simulations with 50% and 90% NCC inhibitions. The predicted Mg^2+^ excretion did not change significantly at 50%, and 90% NCC inhibitions compared with that at 70% NCC inhibition in both male and female rats. This further validates that Mg^2+^ and Na^+^ transport along the DCT is dissociated, as we had observed from our simulation results shown in figure 4, where setting the male NCC activity with the female value did not lower Mg^2+^ excretion significantly (refer to §3.4).

#### 3.5.3. K-sparing diuretics

K-sparing diuretics, amiloride and triamterene, reduced Mg^2+^ excretion in male rats by ~40% [62]. By inhibiting ENaC, these diuretics hyperpolarize the luminal membrane potential and increase K^+^, Ca^2+^ and Mg^2+^ uptake [49–52]. Our model simulations predicted Mg^2+^ reabsorption along the DCT to increase by 8.2% and 8.6% in male and female rats, respectively (figure 5). Females have a higher percentage increase in Mg^2+^ reabsorption because they have higher ENaC activity. These increased reabsorptions decreased male and female Mg^2+^ excretions by 31% and 46%, respectively. In addition, we also performed simulations with 50% and 90% ENaC inhibitions. At 50% and 90% ENaC inhibitions, the predicted Mg^2+^ excretions decreased by 26% and 37%, respectively, compared with baseline Mg^2+^ excretion in male rats. In female rats, the corresponding values were 40% and 54% decreases, respectively. Thus, varying the ENaC inhibition percentage has a significant impact on urinary Mg^2+^ excretion.

## 4. Discussion

Magnesium balance is achieved by the coordinated actions of the kidneys and the intestine. The average daily Mg^2+^ intake for an adult human is around 250–300 mg, of which approximately 50% is absorbed by the intestine [6]. Within the physiological range of Mg^2+^ intake, urinary Mg^2+^ excretion scales approximately linearly with intestinal absorption. Indeed, the kidney’s essential role in maintaining extracellular Mg^2+^ concentration can be seen in its rapid response to changes in serum Mg^2+^: urine Mg^2+^ has been reported to acutely increase as serum Mg^2+^ increases during a 30-min infusion [63].

Although no sex differences have been reported in serum Mg^2+^ in children [64] or adults [65], Mg^2+^ excretion is typically higher in males [66]. This difference may be attributable, in part, to sex differences in kidney structure and function. Renal transporter patterns are poorly characterized in humans; thus, we seek to understand the underlying molecular mechanisms by considering rodents. Veiras *et al*. [14] reported markedly different transport capacities in different tubular nephron segments of male and female rat kidneys. In the proximal tubule, female rats exhibit greater NHE3 phosphorylation and redistribution to the base of the microvilli, where activity is lower, compared with male rats, as well as lower abundance of Na^+^–P_i_ cotransporter 2 (NaPi2), aquaporin 1 (AQP1) and claudin-2 [67]. As a result, the proximal tubule in the female rat reabsorbs a substantially smaller fraction of filtered Na^+^ compared with the male rat [14]. Model simulations indicate that, because Mg^2+^ transport is coupled to Na^+^ transport, the female rat proximal tubule also reabsorbs a smaller fraction of filtered Mg^2+^ compared with the male (see figure 2*a*–*d*).

For many major solutes—for example, Na^+^, Cl^−^ and Ca^2+^—most of their reabsorption occurs along the proximal tubule, where their luminal concentrations remain close to plasma. In contrast, the proximal tubule is responsible for only 15–25% of the filtered Mg^2+^ load, and because proportionally less Mg^2+^ is reabsorbed relative to water, its concentration rises significantly along the proximal tubule (figure 2*e*). For Mg^2+^, most of the reabsorption occurs along the cTAL, and interestingly, none along the medullary thick ascending limb. The activities of NKCC2, K^+^–Cl^−^ cotransporter (KCC) and Na^+^–K^+^–ATPase are higher in female rats. Taken in isolation, this might suggest a higher female capacity to drive Mg^2+^ reabsorption. However, the luminal Mg^2+^ concentration at the cTAL entrance is higher in males compared with females (figure 2*e*), and the male segment has a larger transport area. With these competing factors, our models predict that to attain similar fractional Mg^2+^ reabsorption along the cTAL in males and females, paracellular Mg^2+^ permeability should be significantly higher in females (the model assumes approximately 50% higher).

Downstream of the macula densa, female rats exhibit higher abundance and phosphorylation of NCC, ENaC and claudin-7 [14]. Given the twofold-higher expression of TRPM6 on the apical membrane of the DCT in female mice, the model assumes that TRPM6 channel density is higher in female rats (by twofold). With these sex-specific Mg^2+^ transporter patterns, together with the higher filtered Mg^2+^ load and larger transport areas in males, the models predict fractional Mg^2+^ excretion consistent with reported values, with urinary excretion ~8% higher in male rats compared with females [13].

Having the cTAL and DCT, instead of the proximal tubule (as in the case of Na^+^ and Cl^−^), handle most of the Mg^2+^ transport may give the kidney a better ability to regulate the Mg^2+^ balance. The ability to fine-tune renal Mg^2+^ transport may be particularly crucial because the serum Mg^2+^ level is orders of magnitude lower than Na^+^ or Cl^−^. Renal Mg^2+^ transport is under hormonal control. PTH increases Mg^2+^ reabsorption in both the cTAL and DCT [68]. PTH increases paracellular Mg^2+^ permeability in the cTAL, likely via direct hormonal control of the function and/or expression of tight-junction proteins. PTH also stimulates Mg^2+^ reabsorption in the DCT [69,70], but the molecular mechanisms are unknown. In particular, PTH does not affect TRPM6 gene expression in the kidney [8]. Other hormones, including calcitonin, vasopressin, glucagon, and β-adrenergic agonists, also enhance Mg^2+^ reabsorption in the cTAL and DCT [71].

Compared with males, the female rats transport a larger fraction of the filtered Mg^2+^ along the DCT (figure 2*c*). As a result, females are more sensitive than males to inhibition of TRPM6 (figure 3). This result is consistent with the observation that hypomagnesemia is more prevalent among female patients with diabetes than males [72–74]. Insulin upregulates TRPM6 activity; thus, patients with lower insulin receptor activity are more susceptible to hypomagnesemia [75]. The higher sensitivity of females to inhibition of TRPM6 may partially explain the higher prevalence of hypomagnesemia in female patients with impaired insulin sensitivity and, as such, attenuated TRPM6 activation.

The DCT is the segment responsible for fine-tuning Na^+^ transport and potentially Mg^2+^ as well. Another notable feature of the DCT Mg^2+^ transport is that, unlike the proximal tubule and thick ascending limb, it is decoupled from Na^+^ transport. This may allow females to better adapt their kidney function to conditions under which the electrolyte balance is altered. Two such examples are pregnancy and lactation. During pregnancy, the female body undergoes major adaptations to support the solute and volume demands of the developing fetus and placenta. In the non-pregnant state, almost all Na^+^ and K^+^ intake is excreted. In contrast, net Na^+^ retention begins from early pregnancy [76,77] and drives the large plasma volume expansion that is seen in a healthy pregnancy and is often sustained during lactation. Similarly, there is net K^+^ retention during late pregnancy. The requirements of Mg^2+^ in pregnancy are less well understood. The serum Mg^2+^ concentration has been shown to decrease in pregnancy [78], but this may be due in part to hemodilution. In lactation, human breast milk provides approximately 42  mg/day in 750  ml [78]. In a modelling study, we conducted simulations to demonstrate that the higher distal Na^+^ transporters in a female rat may allow its kidneys to better adapt to the increased electrolyte and fluid demands in pregnancy [79]. Having a proportionally larger distal Mg^2+^ transport capacity may present females with a similar advantage in the altered Mg^2+^ requirements in pregnancy and lactation. A notable challenge is to simultaneously meet the different demands of a number of vital electrolytes. Due to their coupled transport, regulating Na^+^ transporters would affect K^+^ and Mg^2+^ reabsorption as well. How can the kidney selectively retain Na^+^ but not K^+^ or Mg^2+^? For Mg^2+^ this is made possible, in part, by having a nephron segment where Mg^2+^ transport is decoupled from Na^+^, and by having the ability to adjust Mg^2+^ transport in multiple segments (e.g. PTH regulates Mg^2+^ transport along the cTAL, where Na^+^ and Mg^2+^ transport changes together, and the DCT, where changes occur in opposite directions).

To illustrate how renal Mg^2+^ transport is regulated under perturbed conditions, consider Mg^2+^ restriction. When dietary Mg^2+^ decreases, urinary Mg^2+^ excretion rapidly decreases. However, plasma [Mg^2+^] remains unchanged initially, indicating an upregulation in renal tubular reabsorption of Mg^2+^ [80]. Studies have reported increased Mg^2+^ reabsorption across the epithelium of the cTAL (together with increased absorption of Ca^2+^ but not Na^+^ or Cl^−^) [80,81]. Also observed was an increase in both the transcript and protein levels of claudin-16 [82], which is involved in Mg^2+^ reabsorption. These findings support the idea of an adaptive increase in the paracellular pathway for Mg^2+^ transport along the cTAL. Moreover, dietary Mg^2+^ restriction is associated with an increase in Mg^2+^ reabsorption in the DCT [80,83], where both the gene expression and protein expression of TRPM6 increase during Mg^2+^ restriction [8], facilitating the reduction in urinary Mg^2+^ excretion.

In addition to pregnancy, lactation and dietary restriction, Mg^2+^ homeostasis is also altered in diseases such as diabetes [84] and chronic kidney disease [85]. To conduct *in silico* studies of how the kidney adapts in terms of Mg^2+^ transport in these physiological and pathophysiological conditions, the present model can be incorporated into computational models of kidney function for a pregnant rat [79], a diabetic rat [86] and a nephrectomized rat [17]. For better translational value, human kidney models [87] can be expanded to include Mg^2+^. However, to construct accurate computational models, more data are needed that describe the adaptation of Mg^2+^ transporter in these physiological and pathophysiological conditions.

Computational models of renal tubular function developed in the past two decades [15–22] have provided an accurate accounting of solute and water transport and yielded insights into transport pathways, driving forces and coupling mechanisms. Despite these achievements, the limitations of these models primarily include some that stem from the paucity of experimental data and others that are inherent to the model structure (e.g. not considering spatial inhomogeneity within a compartment or intracellular signalling pathways) [88,89]. As can be seen in table 1, many of the model parameters that characterize Mg^2+^ transport have not been measured, especially in the female kidney. To build more accurate sex-specific computational models, we require more experimental studies on the differences in segmental Mg^2+^ reabsorption between male and female rodents. Notwithstanding the uncertainties in some of the model parameters, the present models yield predictions that are consistent with measurements in wild-type and genetically mutated rats (see §3). In terms of model structure, the present models simulate electrolyte and water transport along a superficial nephron, which constitutes only two-thirds of the nephron population in a rat kidney. The remainder of the nephron population is made up of juxtamedullary nephrons whose loops of Henle extend to various depths of the inner medulla. There are differences in the SNGFR, transport area and transporter activities between the two types of nephrons. This study focuses on a superficial nephron model to gain a clearer understanding of segmental Mg^2+^ transport. In future studies, we will develop a kidney model that incorporates both types of nephrons [15] to obtain more accurate predictions of urinary Mg^2+^ excretion rates. We will also investigate the adaptations/dysregulation of renal Mg^2+^ handling in diseases such as Bartter syndrome and Gitelman syndrome, which cause mutations in Na^+^ transporters that are involved in the regulation of Mg^2+^ transport. In addition, some parameters in our model (such as the ratio of TRPM6 channel density between male and female rats) have been taken from experimental studies conducted on mice because corresponding studies on rats were not available. There are several differences between rat kidney and mouse kidney in terms of SNGFR, tubular dimensions and activities of some transporter proteins [90]. Thus, using some mouse parameters for our rat model might affect the accuracy of our predictions to some extent. In the future, if these parameters are available from experimental studies on rats, we will substitute the mouse parameters with them in our model.

## Data Availability

Data and relevant code for this research work are stored in GitHub: https://github.com/Pritha17/Nephron-Mg_Ca_transport and have been archived within the Zenodo repository: [91].
